# Antibacterial and safety tests of a flexible cold atmospheric plasma device for the stimulation of wound healing

**DOI:** 10.1007/s00253-021-11166-5

**Published:** 2021-02-15

**Authors:** Bouke Boekema, Matthea Stoop, Marcel Vlig, Jos van Liempt, Ana Sobota, Magda Ulrich, Esther Middelkoop

**Affiliations:** 1 Association of Dutch Burn Centres, Zeestraat 29, 1941 AJ, Beverwijk, The Netherlands; 2 Burn Center, Red Cross Hospital, Beverwijk, The Netherlands; 3 Department of Applied Physics, Eindhoven University of Technology, Eindhoven, The Netherlands; 4 Department of Plastic, Reconstructive and Hand Surgery, Amsterdam Movement Sciences, Amsterdam UMC, Vrije Universiteit Amsterdam, Amsterdam, the Netherlands; 5 Department of Pathology, Amsterdam UMC, Vrije Universiteit Amsterdam, Amsterdam, the Netherlands

**Keywords:** Dielectric barrier discharge, Skin wound model, Healthy volunteers, *Pseudomonas aeruginosa*, Methicillin-resistant *Staphylococcus aureus* (MRSA)

## Abstract

**Abstract:**

Cold atmospheric plasma (CAP) devices generate an ionized gas with highly reactive species and electric fields at ambient air pressure and temperature. A flexible dielectric barrier discharge (DBD) was developed as an alternative antimicrobial treatment for chronic wounds. Treatment of *Staphylococcus aureus* in collagen-elastin matrices with CAP for 2 min resulted in a 4 log reduction. CAP treatment was less effective on *S. aureus* on dermal samples. CAP did not affect cellular activity or DNA integrity of human dermal samples when used for up to 2 min. Repeated daily CAP treatments for 2 min lowered cellular activity of dermal samples to 80% after 2 to 4 days, but this was not significant. Repeated treatment of ex vivo human burn wound models with CAP for 2 min did not affect re-epithelialization. Intact skin of 25 healthy volunteers was treated with CAP for 3× 20” to determine safety. Although participants reported moderate pain scores (numerical rating scale 3.3), all volunteers considered the procedure to be acceptable. Severe adverse events did not occur. CAP treatment resulted in a temporarily increased local skin temperature (≈3.4°C) and increased erythema. Lowering the plasma power resulted in a significantly lower erythema increase. Good log reduction (2.9) of bacterial load was reached in 14/15 volunteers artificially contaminated with *Pseudomonas aeruginosa*. This study demonstrated the in vitro and in vivo safety and efficacy in bacterial reduction of a flexible cold plasma device. Trial registration number NCT03007264, January 2, 2017

**Key Points:**

*• CAP strongly reduced bacterial numbers both in vitro and in vivo.*

*• Re-epithelialization of burn wound models was not affected by repeated CAP.*

*• CAP treatment of intact skin was well tolerated in volunteers.*

**Supplementary Information:**

The online version contains supplementary material available at 10.1007/s00253-021-11166-5.

## Introduction

The presence of bacteria in a wound can result in delayed healing and a longer hospital stay. Especially burn patients are more susceptible to opportunistic pathogens, such as *Pseudomonas aeruginosa* and *Staphylococcus aureus* (Dokter et al. 2016). Because current therapies in burn care still have limited effects in eliminating bacteria, resistance to antibiotics is increasing, and antimicrobial therapies can hamper wound healing; additional measures are required.

In recent years, cold atmospheric plasma (CAP) devices have been tested clinically as an alternative treatment to reduce bacterial load and support wound healing. Various plasma devices have been shown to be highly effective against a multitude of bacterial species including clinically relevant pathogens and multidrug-resistant bacterial strains, while buildup of resistance against CAP has not been observed (Daeschlein 2018; Zimmermann et al. 2012). Biological surfaces were however more difficult to disinfect (Maisch et al. 2012; Pavlovich et al. 2013b).

CAP devices for biomedical applications can be divided in two categories based on the technology used to produce plasma. In the first type, plasma is created within the device, which is transported to the treatment site via a carrier gas such as argon. The techniques vary from thin plasma needles to large torches. For the second type, plasma is created by dielectric barrier discharge (DBD) devices at the treatment site in ambient air by applying a high voltage. DBD devices use no counter electrode (surface DBD) or use the body as counter electrode (volume DBD). The antibacterial effects of surface DBDs have been shown in several in vitro studies (Boekema et al. 2016; Ehlbeck et al. 2011; Pavlovich et al. 2013a).

CAP devices, predominantly plasma jets, have been applied to chronic wounds to reduce the bacterial load and stimulate wound healing in vivo but with varying degrees of success (Brehmer et al. 2015; Chuangsuwanich et al. 2016; Daeschlein et al. 2015a; Isbary et al. 2012; Isbary et al. 2010; Isbary et al. 2013b; Klebes et al. 2015; Tiede et al. 2018; Ulrich et al. 2015). Application of the volume DBD-based device PlasmaDerm® on human skin biopsies did not result in cell damage (Awakowicz et al. 2009). Safety and efficacy of this device was tested in a clinical study in patients with venous leg ulcers (Brehmer et al. 2015). Plasma treatment resulted in a significant reduction in the bacterial load of the wounds. Although there were no significant changes in wound size between the two groups, the plasma group showed a larger absolute wound size reduction (non-significant).

Performances of plasma jets and other DBDs were similar, and Assadian et al. concluded in their systematic review that use of atmospheric plasma for chronic wounds is safe (Assadian et al. 2019). Only one severe adverse event (SAE) was reported in 9 studies with 268 patients, which was unrelated to plasma (shifted vertebra) (Brehmer et al. 2015). Most reported adverse events (AEs) were minor sensation of burning or heat, tingling or prickling and tautness of skin. These sensations were absent shortly after ceasing plasma treatment.

Unlike plasma jets and other DBDs, the volume DBD in the present study (PLASOMA prototype) generates plasma in the wound in a closed system. The creation of plasma is accompanied by an electric current in the skin and by electromagnetic fields that can stimulate blood vessel formation and cell proliferation (Li et al. 2012). The volume DBD has been tested in 20 patients with diabetic foot ulcers (DFU) (Peters et al. 2017). Wounds received 10 treatments in 2 weeks with 3× 20 s plasma and 2× 20-s intervals with plasma off. Bacterial load of *S. aureus* was significantly reduced after CAP application. Fifty-five percent of the patients reported transient and mild AE; SAE related to the treatment did not occur. However, many of these patients probably had limited feeling in their feet due to neuropathology associated with DFU (Volmer-Thole and Lobmann 2016). In burns on the other hand, acute pain is an important complication. Burn patients can experience procedural pain during wound care procedures such as during dressing changes, which is difficult to control (van Loey, personal communication, February 11, 2020). In addition, burn patients suffer background pain, which varies greatly in intensity, is experienced at rest and can be caused by inflammation. Therefore, before exposing burn patients to possible additional pain caused by plasma treatment, we tested safety and efficacy on intact skin of healthy human volunteers. We also tested safety and efficacy of the PLASOMA prototype in vitro on bacteria and dermal tissue to answer the question: Can bacterial load be reduced with a flexible volume DBD device without causing damage to cells in wounds?

## Materials and methods

### Plasma device

**Fig. 1:**
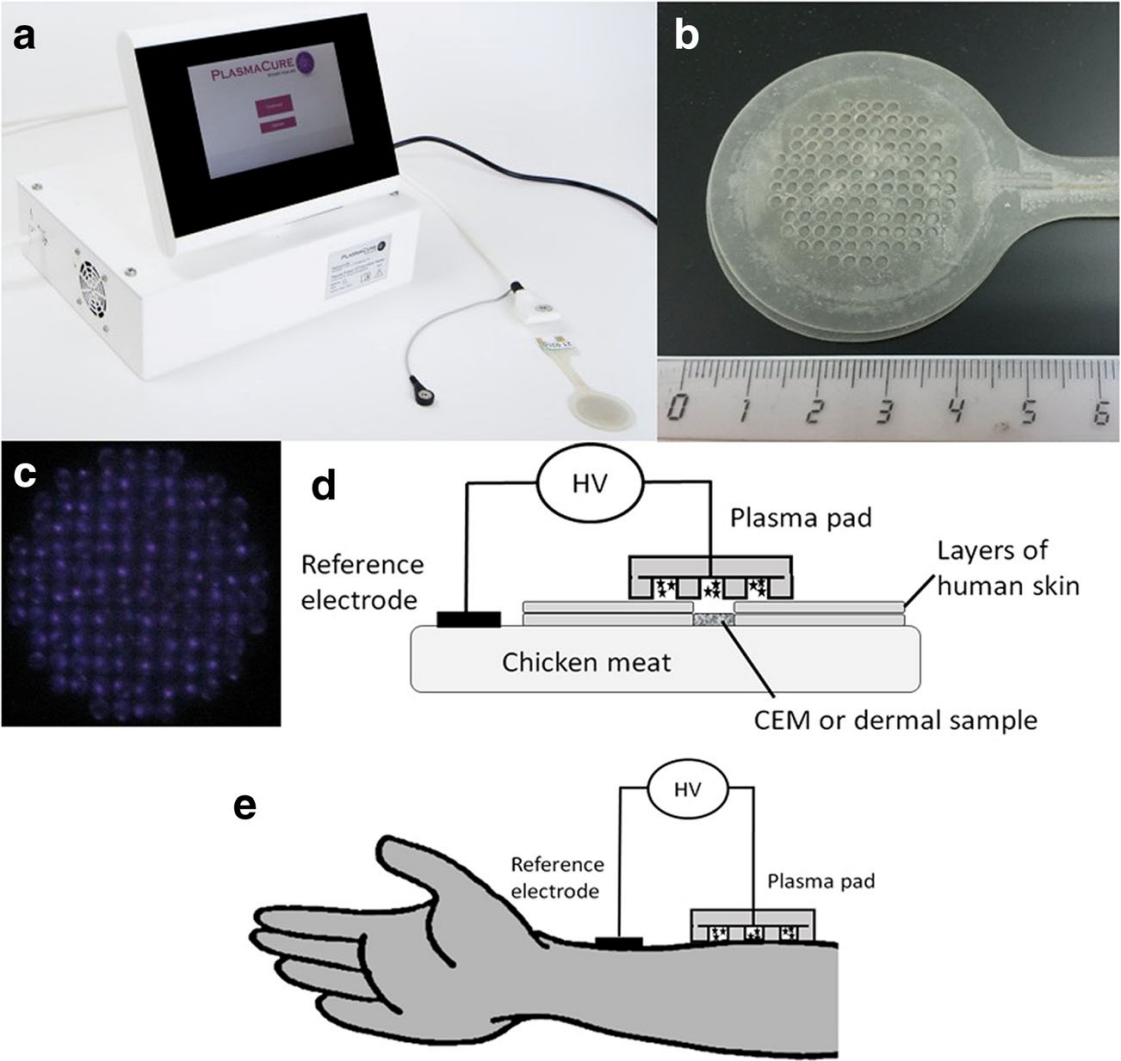
**a** The plasma device consisting of the plasma driving unit (plasma pulser) and plasma pad; **b** plasma pad with scale in cm. Plasma pad showing the side with prefabricated holes that is in contact with the skin; **c** plasma is generated in the small holes; **d** schematic diagram of device for the treatment of samples on chicken meat as a support layer; layers of human skin (0.7 mm) were used to increase the distance between sample and plasma; **e** schematic diagram of device during treatment of healthy volunteers. HV, high voltage; CEM, collagen elastin matrix

Plasma power source and pads were provided free of charge by Plasmacure (Nijmegen, the Netherlands) (Fig. 1). Plasma is generated in pads made of flexible material. For the reference electrode, a standard ECG patch was used (red dot, 3M Health Care, Germany). The driving unit can only operate when proper connection of the pad is detected. Plasma is only created when the pad makes contact with the subject’s body. To further protect the subject, plasma treatment is stopped automatically in case a threshold current or voltage is reached. Plasma was generated at a high and a low power setting.

### Ozone measurements

Ozone density was determined by absorption spectroscopy at 266 nm, using an Nd:YAG laser (ULTRA CFR Nd:YAG Laser System) coupled through optical fibres and collimated using quartz lenses (Gorshelev et al. 2014). The pad was placed on a steel mesh with openings of approximately 1 mm that was grounded, creating plasma between the pad and the mesh. The light path was positioned approximately 2 mm above the mesh, in a closed cylinder 30 mm in diameter (the size of the active area of the pad) and 4-mm thick. The absorption path was therefore 30-mm long. Plasma was generated at high power setting, with 4% difference in the voltage amplitude.

### Human skin

Human skin was obtained from healthy donors undergoing dermolipectomy or from deceased donors via the Euro Tissue Bank, after obtaining consent according to institutional guidelines. Split-thickness skin grafts (0.7 or 1 mm thickness) were harvested using a dermatome.

### Bacterial culture and quantification

*P. aeruginosa* (strain PAO1, ATCC BAA47, culture collection NCCB 2452, obtained from J. Tommassen, UU, the Netherlands) and *S. aureus* (Methicillin-resistant *S. aureus* (MRSA), strain LUH14616, culture collection NCCB 100829, obtained from P. Nibbering, LUMC) were routinely cultured on Luria-Bertani broth (LB, Invitrogen, Paisley, UK) agar at 37°C. Bacteria from a proliferating or logarithmic culture in 5 ml LB were diluted in 0.85% NaCl to the required colony forming units (CFU)/ml, based on OD_600_. To determine bacterial numbers, collagen-elastin matrices, dermal samples or the viscose ends of swabs were placed in a vial with 1 ml PBS and a metal bead and were placed in a TissueLyser LT (Qiagen, Venlo, The Netherlands) for 4 min at 45 Hz (Boekema et al. 2013). Bacterial suspensions were serially diluted and plated on agar plates: LUH14616 on LB agar and PAO1 on selective Pseudomonas Isolation Agar (Oxoid) plates supplemented with 0.25% Cetrinix (Sigma, St. Louis, MO) (PIA-CN).

### Plasma treatment in vitro

Bacterial solutions (10 μl, 10^5^ CFU) were pipetted on dry porous collagen-elastin matrices (CEM, Ø 15 mm, 1 mm thickness); CEM prewetted with 100 μl 0.85% NaCl or on skin (Ø 15 mm, 1 mm thickness, dermal side). These samples were placed on agar, metal or chicken meat to which the reference electrode was connected. Chicken meat represented tissue that is present underneath wounds. To mimic the in vivo situation, ex vivo human skin with a hole (Ø 15 mm) was placed around the samples (Fig. 1d). Plasma pads were placed on top of the samples. Layers of the skin were added to increase the distance between CEM and plasma pad from 0 to 0.7 mm and 1.4 mm to simulate deeper wounds. After plasma treatment at low power setting, samples were processed for bacterial quantification. The log reduction (LR) was calculated: LR = log CFU before – log CFU after treatment.

Similarly, human skin biopsies (Ø 15 mm, 1 mm thickness) were treated. Three punch biopsies (Ø 4 mm) were taken for viability assessment.

### Viability assays

To estimate tissue viability after treatment, MTT assays were performed (Coolen et al. 2010). Immediately after plasma treatment of dermal samples at low power setting, biopsies (4 mm) were taken and transferred to 24-well plates with 2 ml Dulbecco’s Modified Eagle Medium (DMEM) supplemented with 10% (v/v) foetal calf serum (FCS), 1% (v/v) penicillin/streptomycin (P/S) and 1% GlutaMAX (all from Gibco, Paisley, UK) further referred to as fibroblast medium (FBM), containing 2 mg/ml MTT (3-(4.5-Dimethylthiazol-2-yl)-2.5-diphenyltetrazolium bromide). Samples were incubated with MTT medium for 3 h at 37°C, 5% CO_2_. Formazan was dissolved by shaking skin samples in the presence of 1 ml DMSO and a metal bead during 4 min at 50 Hz (TissueLyser). The resulting supernatant was quantified in a spectrophotometer (OD_560_-OD_650_, SpectraMAX, Molecular Devices). Results were expressed relative to untreated controls (in %). For repeated treatments, skin samples were incubated in-between treatments in FBM at 37°C, 5 CO_2_ for 24 h.

### Burn wound model

To determine the effect of plasma on wound healing, an ex vivo human skin model was used (Boekema et al. 2013). Split-thickness skin grafts (0.7 mm thickness) were cut into 1.25 cm^2^ pieces. Burn wounds were created with a copper device (2 × 10 mm) attached to a soldering iron (95°C, applied for 10 s). This would correspond to a full-thickness burn. The burned wound models (BWMs) were placed epidermis up on stainless steel grids and were cultured air exposed at 37°C with 5% CO_2_ using DMEM/ Ham’s F12 (3:1) supplemented with 2% (v/v) P/S, 2% (v/v) FCS (Gibco), 1 μM hydrocortisone, 1 μM isoproterenol, 0.1 μM insulin, 1 μM L-carnitine, M L-serine, 1 μM DL-alpha-tocopherol, 130 μg/ml ascorbic acid, a lipid supplement containing 25 μM palmitic acid, 15 μM linoleic acid, 7 μM arachidonic acid (all from Merck KGaA) and 24 μM BSA (Thermo Fisher Scientific).

Metal grids with BWMs were placed on a metal surface, which was connected to the reference electrode. Plasma treatments at low power setting were applied for 1 or 2 min on the BWMs, and treatments were repeated on days 3, 7 and 10. Controls consisted of untreated BWMs. Samples were kept in culture for up to 2 weeks. Twenty-four hours before fixation of BWMs in kryofix (50% ethanol and 3% PEG300), 20 μM 5-bromo-2-deoxyuridine (BrdU) (Merck KGaA) was added to the culture medium.

### Histology

Skin samples were processed for paraffin embedding. Sections (5 μm) were deparaffinized and rehydrated for haematoxylin and eosin (H&E) staining, using standard techniques. Sections from BWMs were stained for BrdU (MP Biomedicals, Illkirch, France) (Coolen et al. 2010). Antigen retrieval consisted of incubation in 10 mM sodium citrate solution for 20’ at 65°C (γH2AX) or in 2 M HCl for 30’ at room temperature followed by 0.1 M Borax pH 8.5 (twice 5’) and 7’ in 0.5 % Triton-X 100 in PBS (BrdU). Brightvision poly HRP-Anti-Mouse IgG and Bright-DAB Solution (Immunologic, Duiven, the Netherlands) were used for visualization. The newly formed epidermis was measured with digital image analysis (NIS Elements Ar software, Nikon, Amsterdam, the Netherlands). To monitor double strand breaks, sections were stained with an antibody against phosphorylated γH2AX (MA1-2022, Thermo Fisher Scientific) followed by Alexa Fluor® 555-conjugated goat-anti-mouse antibody (Molecular Probes, Thermo Fisher Scientific) and DAPI (Sigma) (Isbary et al. 2013a).

### Trial design and demographics

We performed an interventional safety study with intra-individual comparison. The study protocol was approved by the regional ethics committee of Noord-Holland, the Netherlands (reference number M016-046), registered at clinicaltrials.gov (registration number NCT03007264, January 2, 2017). The study followed the tenets of the Declaration of Helsinki (52nd World Medical Association General Assembly, Edinburgh, Scotland, October 2000). A total of 25 healthy subjects were included from the general public; informed consent was obtained. Exclusion criteria were skin disease, infected wounds, implanted electrical medical devices, (possible) pregnancy, life-threatening cardiac conductivity abnormality and active malignancy. A research nurse assisted the procedures. Sixty percent of volunteers were male, average age was 58 ± 12 years and Fitzpatrick skin type 3 was most prevalent (56%) (Table S1). Subjects were randomly divided over 3 groups: (A) plasma at high power, (B) bacteria and plasma at high power and (C) bacteria and plasma at low power.

### Pain measurement

Pain scores were determined on a numerical rating scale (NRS) of 0–10. A pain score less than 4 is considered acceptable pain (de Jong et al. 2015), a pain score between 4 and 7 is moderate-severe pain and above 7 severe pain. Subjects were asked to qualify the experienced pain as acceptable or not.

### Skin temperature

Thermal images (as shown in Figure S1) were produced using the FLIR ONE camera (FLIR Systems, Inc., Wilsonville, OR, USA) attached to an iPad mini (Apple Inc., Cupertino, CA, USA). The mean temperature of the reference area (wrist) was subtracted from the mean temperature of the area of interest (inner forearm) and was expressed as dT (°C) (Jaspers et al. 2017a). Subsequently, dT at baseline (before treatment) was subtracted from dT after treatment yielding a change in temperature due to treatment.

### Skin colour measurement

Skin colour was measured by means of the DermaSpectrometer® (Cortex Technology, Hadsund, Denmark). This reliable narrowband spectrometer generates an erythema index based on the differences in light absorption of red haemoglobin (Draaijers et al. 2004).

### Skin barrier function

Skin barrier function was evaluated by measuring TEWL (Tewameter® TM300 probe, Courage & Khazaka GmbH, Cologne, Germany). TEWL estimates the flux density of evaporated water from the skin surface (Tagami and Yoshikuni 1985) and is considered an important physiological characteristic to assess the function of the skin barrier (Rogiers 2001).

### Treatment of volunteers

Baseline measurements were performed on both inner forearms for all subjects (Figure S1). Water resistant pen was used to mark the areas for the pads on both arms.

For group A, plasma pads were placed on both arms and were secured with Microfoam surgical tape (3M); ECG patches (3M red dot, 3M) were placed on the right arm next to the pad to connect the reference electrode. Plasma treatments were applied (high power setting) on the right arm: 3 times for 20 s with plasma on separated by 2 intervals for 10 s with plasma off. The contralateral arm was treated exactly the same except plasma was not applied. One pad was used per arm, and pads were not changed between intervals. Pads and reference electrode were removed directly after treatment. Measurements were done immediately after treatment and after 30 min.

For group B and C, 15 μl of bacterial suspension (PAO1, 10^7^ CFU/ml) was applied on the area of interest on both arms and left to dry for a few minutes. Plasma pads and ECG patches were placed as in group A. Plasma treatments were applied 3 times for 20 s with plasma separated by 2 intervals for 10 s with plasma off, at high (group B) or low power setting (group C). Pads and reference electrode were removed directly after treatment, and thermal images were taken instantly and after 30 min. The scrub wash method (Taylor et al. 2003) was used to collect all surviving bacteria, using a swab and 3 times 0.5 ml Dulbecco’s Phosphate-Buffered Saline (PBS, Gibco®; Thermo Fisher Scientific, Etten-Leur, the Netherlands), 0.1% Triton X-100 (Lademann et al. 2012; Li et al. 2013). Skin was disinfected with 70% alcohol and dried. Measurements for erythema and TEWL were done immediately after this and after 30 min. The log reduction (LR) per subject was calculated:

LR = log CFU before – log CFU after treatment.

All subjects were contacted after approximately 1 week to check for any potential adverse events.

### Statistics

Statistical analysis was performed with SPSS (Version 16.0 for MS Windows, SPSS Inc., Chicago, IL). The Mann-Whitney *U* (MWU) test was used to determine significant differences due to CAP treatment or between groups. Wilcoxon matched-pairs signed-rank test (WMP) was used for paired samples (e.g. intra-individual comparison). Results of MTT assays were expressed relative to their respective untreated controls before averaging to circumvent donor variations. Volunteer characteristics sex and skin type were compared using the chi-square test for categorical data.

## Results

A flexible DBD plasma pad was developed to enable treatment of curved surfaces. Here we report the bactericidal and safety effects of in vitro tests. An interventional study on intact skin of human volunteers was conducted to study safety and efficacy in vivo.

### Bactericidal efficacy in vitro

Initial tests were performed with bacteria spread on agar plates. These tests showed that the volume DBD pad inactivated bacteria effectively after short treatment times (< 2 min, data not shown). To simulate the wound environment and for a better quantification of the bactericidal effect, we tested the plasma pads on bacteria in a 3D-collagen/elastin matrix (CEM, Ø 15 mm, 1 mm thickness). Collagen and elastin are major components of the dermis. CAP treatment of bacteria in dry CEM also resulted in high reduction (log 4 reduction) after <2 min treatment (data not shown).

**Fig. 2:**
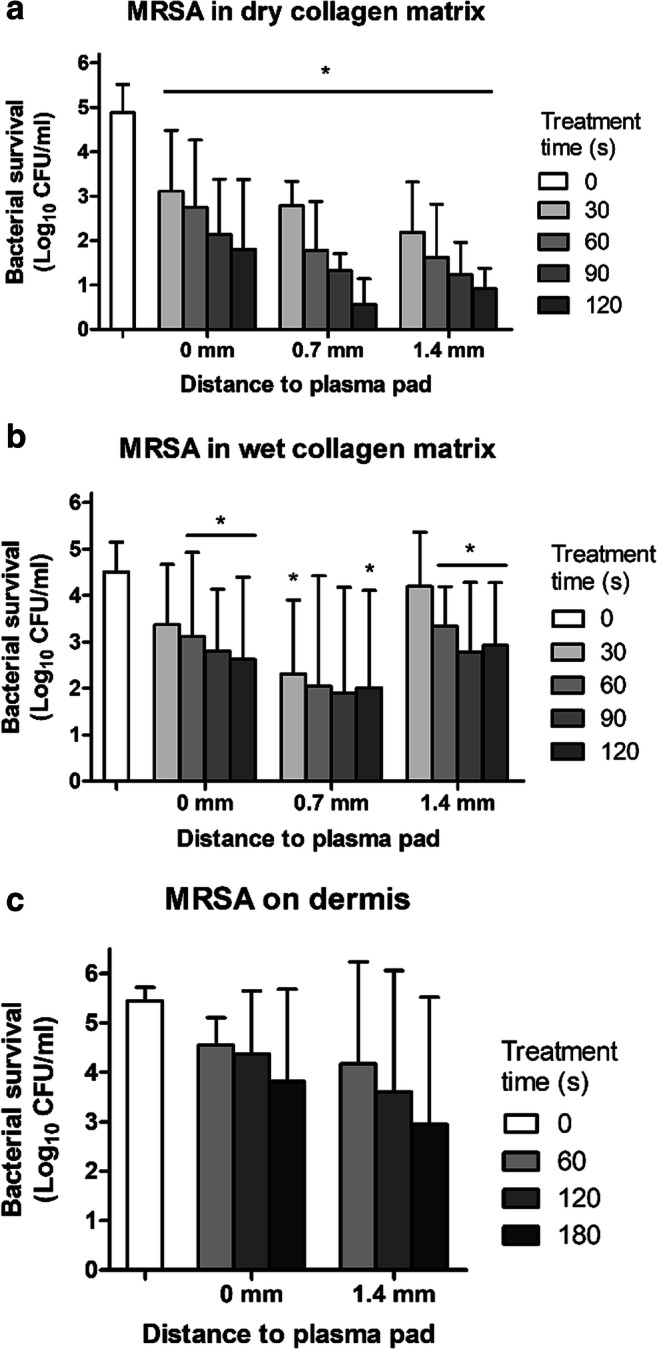
Surviving bacteria after plasma treatment of 10 μl MRSA strain LUH14616 (**a**) in dry CEM (*n*=6 in duplicate), (**b**) in wet CEM (n=5 in duplicate) or (**c**) on human dermis (*n*=3–6 in duplicate). All samples were placed on chicken meat; human skin was placed around them. Distance between plasma pad and tissue was increased by adding layers of skin surrounding the CEM. Mean values and standard deviation are shown. * Significant difference compared to untreated control samples (0 s), *p*<0.05 (MWU).

To mimic the in vivo wound situation even more, CEMs were placed on chicken meat, while human skin was placed around the CEM (see Fig. 1e). Plasma treatment reduced the bacterial load from log 4.7 to <log 1 when bacteria were added to dry CEM (Fig. 2a). Bacterial reduction was dependent on plasma treatment time. In larger wounds with varying depths, the plasma pad might not always make contact to the entire surface of the wound. This was simulated by adding layers of skin (0.7 mm thickness) to increase the distance between CEM and plasma pad (Fig. 1d). The plasma pads were equally effective on bacteria at 0 to 1.4 mm (Fig. 2a). Because the wound environment is moist, the effect of a higher water content was tested. When plasma was applied to bacteria present in CEM with a higher water content (110 μl), the bactericidal effect was significant in specific conditions but was reduced in general (Fig. 2b). A similar approach was used to determine the bactericidal effect of plasma on samples of human dermis (Ø 15 mm). The bactericidal effect was however strongly reduced on dermal samples (Fig. 2c).

### Safety tests in vitro

**Fig. 3:**
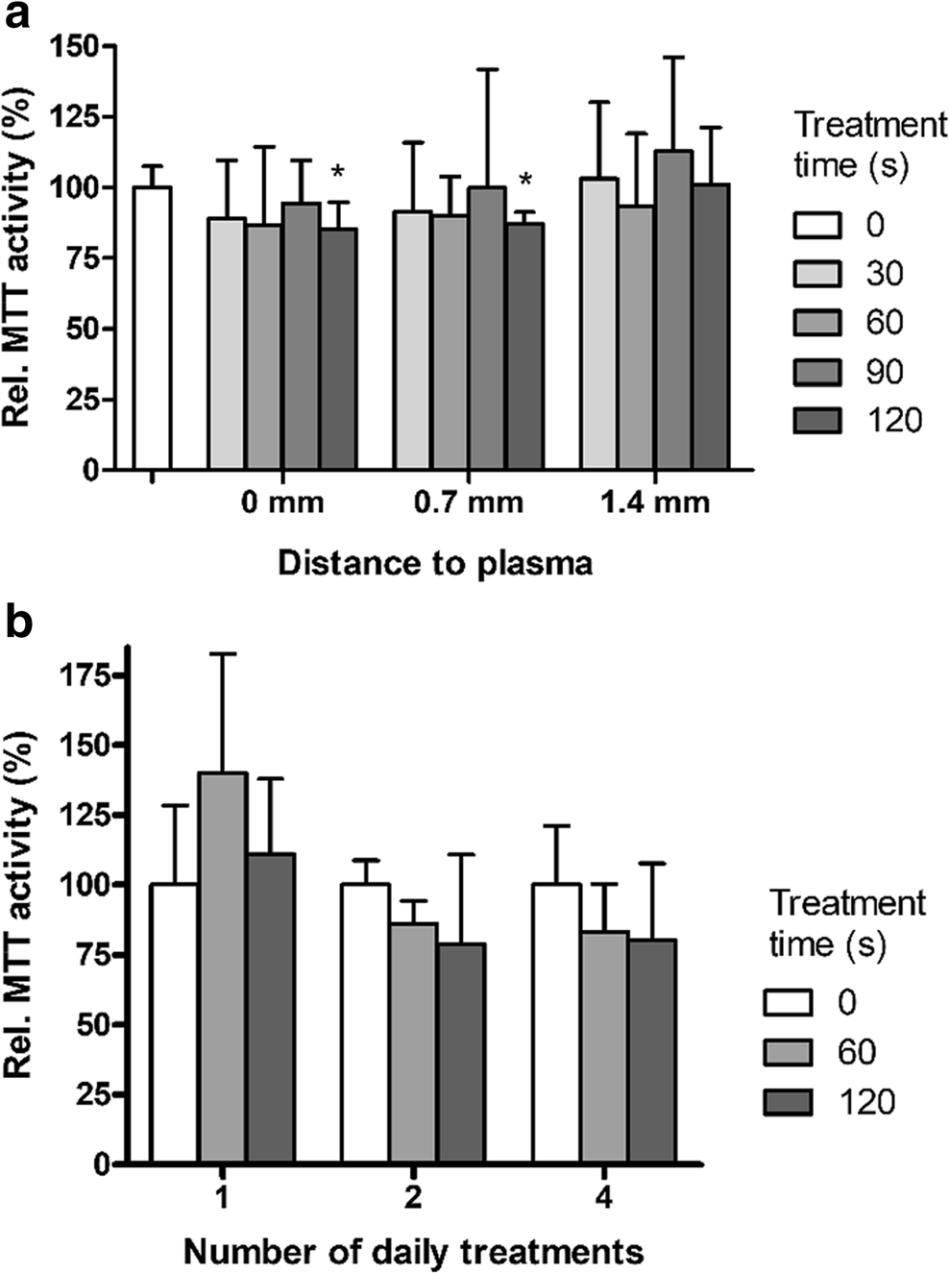
**a** Effect of CAP on cellular activity of human dermis (Ø 15 mm, 1 mm thickness) placed on chicken meat. Distance between plasma and tissue was increased by adding layers of skin surrounding the dermal samples. Plasma was applied for the indicated times. Cellular activity was measured with MTT in 3 punch biopsies (Ø 4 mm) and is expressed as relative to the untreated controls (in %). Mean values and standard deviation are shown (*n*= 4–7 in duplicate). **b** Effect of repeated CAP treatment on cellular activity of human dermis measured with MTT. Plasma was applied on consecutive days for the indicated times in the *x*-axis. Dermal samples were incubated in FBM at 37°C, 5% CO_2_. Mean values and standard deviation are shown (*n*= 3–5 in triplicate). * Significant difference, *p*<0.05 (WMP)

For safety assays, a similar setup was used. Dermal samples were placed on chicken meat, while human skin was placed around the sample to mimic the in vivo situation (Fig. 1e). Dermal samples were chosen because in wounds the epidermis is (mostly) absent. More layers of skin (0.7 mm thickness) were added to increase the distance between dermal sample and plasma pad. Samples were CAP treated, and cellular activity was determined (MTT) as a measure of cell viability (Fig. 3a). Viability of these samples was not reduced by CAP treatments of up to 90 s for all tested distances to the plasma. CAP treatment for 120 s slightly reduced activity at 0 and 0.7-mm distance (*p*<0.05).

Since potentially multiple treatments will be given to patients, we tested repeated treatments on dermal samples. Dermal samples received plasma treatments of 60 or 120 s on consecutive days and were incubated in-between in medium at 37°C, 5% CO_2_. Viability was decreased after 2 and 4 daily CAP treatments to 80% (non-significant, Fig. 3b).

**Fig. 4:**
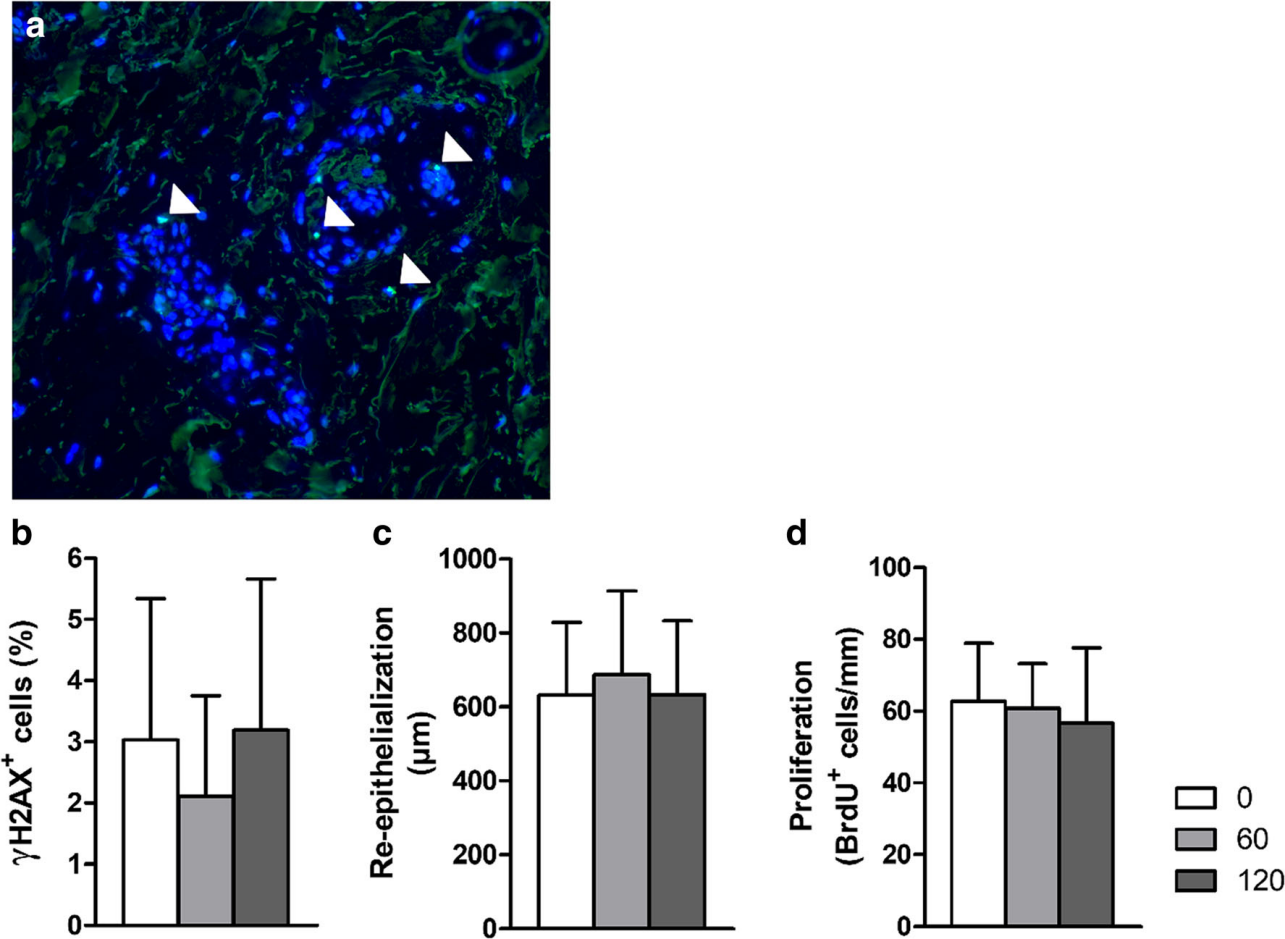
**a** Example of dermal sample after plasma treatment, stained for γH2AX. Positive nuclei are indicated (arrow head); bar is 50 μm. **b** Quantification of the relative number of cells (%) positive for γH2AX after plasma treatment of dermal tissue (*n*=4–5 in duplicate). **c** Re-epithelialization and **d** proliferation of keratinocytes in BWM after 2 weeks of culture. Plasma was applied four times in 10 days. Mean values and standard deviation are shown (*n*=6 for 0 and 120 s, *n*=4 for 60 s, all in duplicate). Significant differences were not observed

Because plasma might affect DNA integrity (Wu et al. 2013), we evaluated phosphorylation of γH2AX, which is involved in repair of double-strand breaks (Kuo and Yang 2008). Dermal samples were plasma treated for 60 or 120 s and were processed for immunohistochemistry (Fig. 4a). The number of positive nuclear foci were counted and corrected for the total number of nuclei. Plasma treatment did not show more γH2AX positive cells compared to untreated control samples (Fig. 4b).

To monitor the effect of plasma treatment on wound healing, we treated burn wound models consisting of ex vivo human skin for 60 or 120 s, for four times in 10 days. Repeated plasma treatment did not affect re-epithelialization or the number of proliferative keratinocytes in the neo-epidermis (Fig. 4c).

**Fig. 5:**
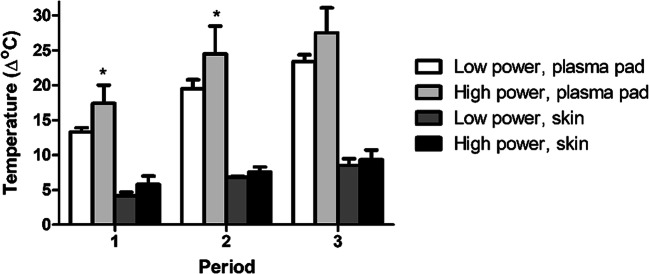
Relative increase in temperature due to plasma treatment. Ex vivo skin was plasma treated for 3× 20 s and 2 intervals of 20 s with plasma off. Thermal images of pad and the skin were made directly at the end of each period of 20 s of plasma on. Shown is the average temperature at the treatment area minus average temperature at the reference area in °C. * Significant difference compared to low plasma power, *p*<0.05 (MWU)

Although in vitro tests demonstrated safety of CAP for tissues and wound healing, heat generated by CAP might pose a problem when treating human subjects. We measured the temperature of ex vivo skin (placed on metal) and plasma pad directly after CAP treatment. To limit the buildup of heat, the plasma treatment was divided in three periods of 20 s separated by intervals of 20 s with plasma off. Temperature of the plasma pad increased with treatment time (Fig. 5). As a result, the average ex vivo skin temperature increased with 9°C after 3 times 20 s of plasma treatment. To investigate whether increasing plasma power affects warming of the skin, two settings were tested. Although the high plasma power setting significantly increased temperature of the plasma pad after the first and second period, it did not significantly affect the increase in skin temperature.

### Ozone density

**Fig. 6:**
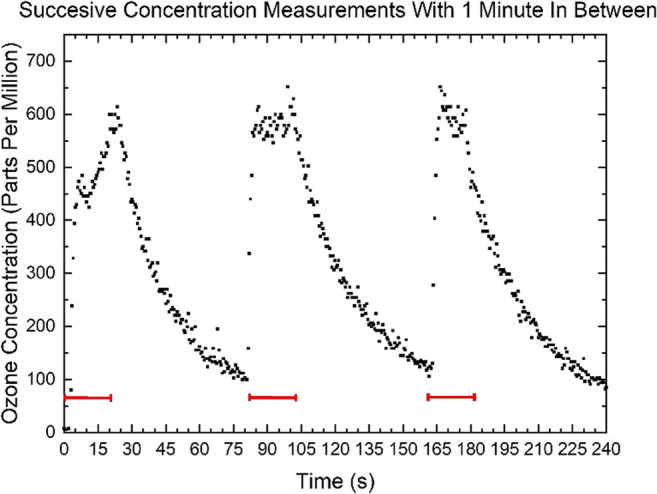
Concentration of ozone produced by the plasma pad during three successive periods of 20 s (plasma on) separated by 60-s intervals (plasma off). The red bars indicate when the plasma is on

Ozone density measurements were performed using plasma treatment divided in three periods of 20 s separated by 2 intervals of 60 s with plasma off. During successive plasma periods of 20 s, ozone is generated at a concentration up to 600 ppm (Fig. 6). The ozone concentration drops between successive periods, but the gas mixture requires more than 60 s to revert back to its original state before the treatment. Successive measurements with shorter plasma off intervals showed the same trend of decrease in ozone density between periods and no increase in the peak ozone concentration.

### Safety tests in healthy volunteers

Because good results were obtained with the in vitro tests of CAP treatment, we conducted a clinical trial. Since CAP may elicit some pain, we tested safety of CAP in healthy volunteers. A total of 25 healthy volunteers divided over 3 groups were included in this study; the demographics are shown in Table S1. Data generated from two subjects in group A were excluded from further analysis because plasma had not been produced, based on skin temperature and erythema. In group A, plasma was applied on the right arm at high power, and skin parameters were measured directly and 30 min after treatment. In group B and C, a bacterial suspension was placed on both arms. Plasma was applied on the right arm at high (group B) or low power (group C). After collecting the surviving bacteria from both arms, skin parameters were measured directly and 30 min after plasma treatment.

The following transient AEs (grade 1) were reported by the subjects: warmth sensation (all subjects) and itching until one day after treatment (1 subject in group A). SAEs did not occur.

The primary outcome pain estimated with NRS directly after CAP treatment was on average 3.3 (Table 1). All subjects indicated that the pain experienced during treatment was acceptable. Although pain scores were slightly lower in group C, pain scores were not significantly different between groups (*p*<0.05, MWU). Pain scores were 0 after 30 min for all subjects.

**Table 1:** Primary outcome pain was assessed with NRS

Group	N	Pain directly after plasma	Range	Tolerable	Pain 30 min after plasma
A	8	4.0 ± 2.4	0–7	100%	0
B	8	3.3 ± 1.7	1–6	100%	0
C	7	2.4 ± 1.8	0–5	100%	0
All	23	3.3 ± 2.0	0–7	100%	0

Mean ± standard deviation, range and tolerability are shown

**Fig. 7:**
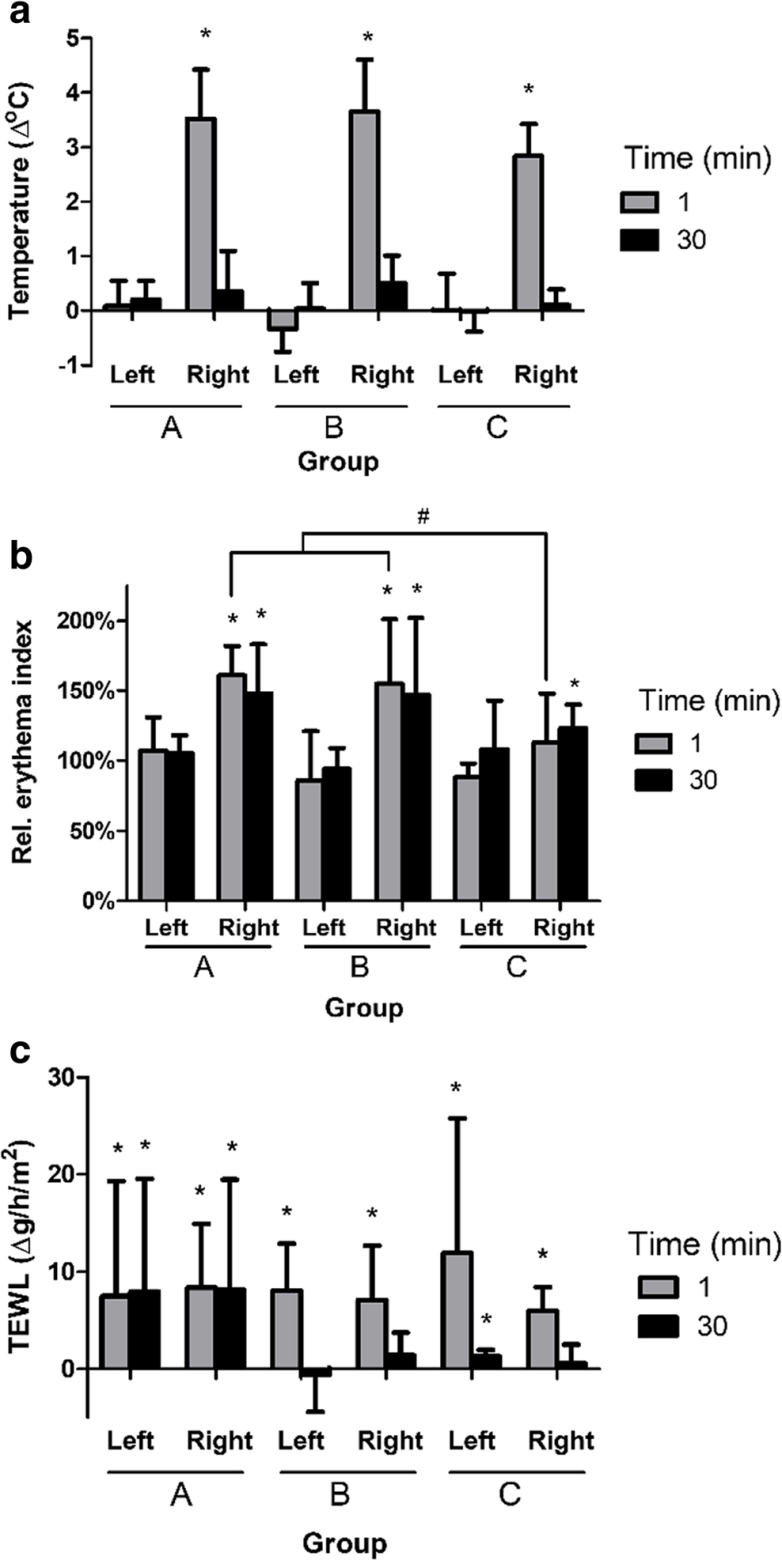
**a** Relative skin temperature due to plasma treatment. Shown is the average skin temperature at the treatment area minus average skin temperature at baseline in °C. **b** Erythema index relative to baseline. **c** TEWL values relative to baseline. Mean values and standard deviation directly (1) or 30 min after plasma treatment are shown. Left was the untreated arm, and right was the plasma treated arm. * Significant difference versus baseline, *p*<0.05 (WMP). # Significant difference between indicated groups, *p*<0.05 (MWU)

Baseline values were recorded on both inner volar arms. The mean absolute skin temperature at baseline was 32.0 ± 3.8°C. CAP treatment significantly increased local skin temperature by on average 3.4°C compared to baseline in all groups (Fig. 7a). After plasma application, skin temperature decreased and was similar to the reference area after 30 min. No changes were observed in skin temperature on the left arm. Although mean skin temperature directly after CAP was slightly lower in group C, significant differences between groups were not noted.

Plasma treatment also significantly increased the redness of the skin (Fig. 7B). Erythema index at baseline was on average 11 and increased with approximately 52% directly after plasma treatment in groups A and B (*p*<0.05, WMP). The lower power setting in group C resulted in a significantly lower erythema index (8% increase) compared to the high power setting (*p*<0.05, MWU). Thirty minutes after treatment, erythema index of the treated arm was still significantly increased in all groups (*p*<0.05, WMP). No changes were observed in erythema index on the left arm.

TEWL measures the evaporation of water from the skin, which characterizes the barrier function of the stratum corneum. TEWL was significantly increased directly after treatment in all three groups (Fig. 7C). Because TEWL was also increased on the untreated contralateral arms (left), TEWL increase on the CAP-treated right arm was regarded as not related to plasma. Thirty minutes after the pads were removed, TEWL was still significantly increased in groups A (both arms) and C (left arm) (*p*<0.05, WMP). Significant differences between groups were not noted. TEWL after 30 min was lower on both arms in groups B and C than in group A, which was most likely the result of the swabbing and cleaning procedure used to determine bacterial survival in groups B and C.

### Antibacterial effect in healthy volunteers

**Fig. 8:**
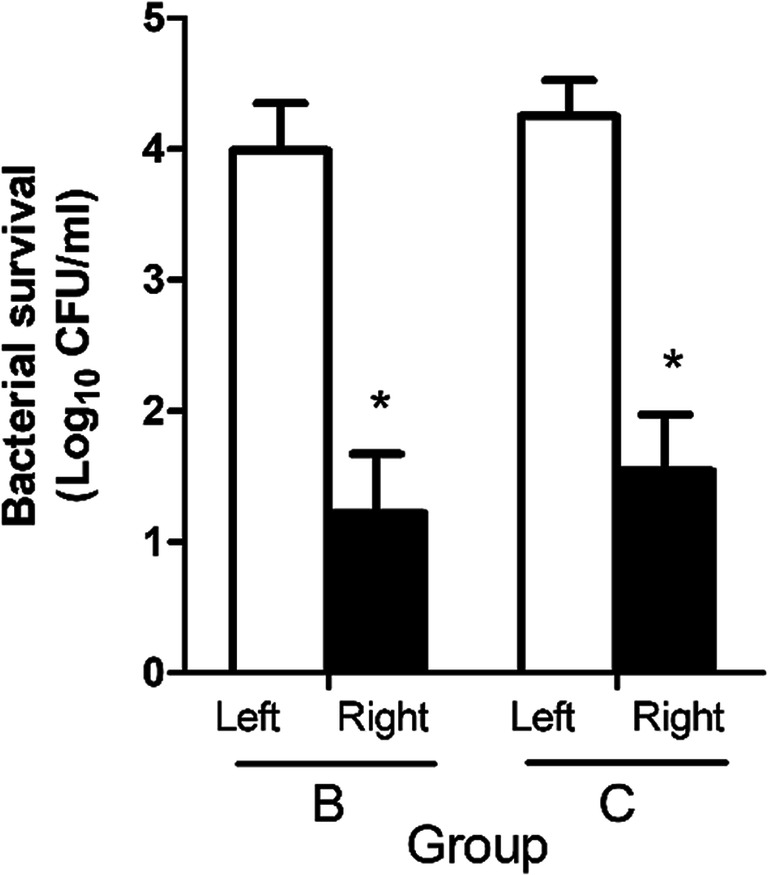
Bacterial survival of *P. aeruginosa* PAO1 in Colony Forming Units (CFU), determined directly after plasma treatment for groups B and C. Mean values and standard deviation are shown. Left was the untreated arm, and right was the plasma treated arm. * Significant difference of plasma treated (right) versus untreated control (left), *p*<0.05 (WMP).

To study the bactericidal effect of CAP, the skin of both inner forearms of 15 volunteers (groups B and C) was contaminated with *P. aeruginosa*. CAP was only applied to the area on the right arms. Surviving bacteria were collected from both arms by scrub wash and counted after plating. Data from one subject were excluded because of low bacterial survival on the untreated left arm. CAP significantly reduced the number of bacteria in both groups (Fig. 8). The mean log reduction was 2.9 and was not significantly affected by plasma power setting.

## Discussion

In this study, we have shown the bactericidal property and safety of a flexible CAP device, both in vitro in wound models and in healthy volunteers on intact skin. Short plasma treatments were able to greatly reduce bacterial load and did not cause DNA damage or reduce cellular activity or wound healing.

The fast bactericidal effect of CAP was reduced by moisture and nature of the treated target such as skin vs. CEM (Fig. 2), which has been observed before. The skin was more difficult to disinfect by CAP treatment than silicon or rubber (Pavlovich et al. 2013b). Humidity has been shown to be a critical factor for plasma inactivation of bacterial spores (Patil et al. 2014). The species responsible for bacterial killing (i.e. reactive oxygen or nitrogen species) might have reacted with other components present such as water and proteins of the skin.

One of the reactive species involved in CAP-mediated bacterial killing is ozone. Up to 600 ppm ozone was locally produced by the DBD plasma pads (Fig. 6). In contrast, jet-based plasma treatments exhibit low ozone production levels (≈2 ppm) (Zhang 2015; Zhang et al. 2013). Ozone has been reported to correlate with bacterial killing by a DBD device (Pavlovich et al. 2013a), and ozone was very effective against bacteria at 3–20 ppm in saline solution (Burgassi et al. 2009; Zeng et al. 2020), while >100 ppm was required when bacteria were present in diluted blood plasma (5–10%) (Burgassi et al. 2009). High concentrations of ozone can however be a potential safety issue such as irritation of skin, eyes, and mucous membranes of the respiratory tract. Ozone may cause drowsiness, dizziness, headache, and fatigue. Limits for exposure via inhalation to ozone vary between 100 (guideline WHO) and 120 (guideline European Union) μg/m^3^ for 8 h per day. The PLASOMA prototype produces ≈3 μg ozone per 20-s period, which does not pose a problem.

Single treatment with CAP for 120 s slightly reduced the cellular activity in dermal samples to 86% (Fig. 3), while 70% is the cut off for moderate toxicity (ISO10993-5 2015). Similar tests with different types of DBD devices have been performed on cells or skin in vitro (Awakowicz et al. 2009; Boekema et al. 2016; Dijksteel et al. 2020; Heuer et al. 2015; Isbary et al. 2013a; Laurita et al. 2017; Maisch et al. 2012) or in vivo (Rajasekaran et al. 2011; Wu et al. 2013), and the results on cellular activity, inflammation or DNA damage were found to be within safe limits. In addition, repeated CAP treatment with the PLASOMA prototype did not significantly lower activity of cells in dermal samples (Fig. 3) and did not affect epidermal regeneration or DNA integrity in human burn wound models (Fig. 4), which underscores the safety of CAP treatment. It is important to note that in these samples there is no blood flow or influx of cells as in the human body, which can affect heat transfer and healing rate. Repeated or extended exposure to CAP might result in an accumulation of cell damage. In the literature, repeated in vitro CAP treatments on tissue samples or cell cultures have not been described. However, in animal and clinical studies, repeated CAP treatments have been shown to be safe (Kisch et al. 2016b; Metelmann et al. 2012; van der Linde et al. 2017). Taken together, in vitro CAP treatment with the PLASOMA prototype for 1 or 2 min was effective and safe.

A systematic review showed that plasma treatment in general was safe (Assadian et al. 2019). Treatment with the here tested PLASOMA prototype was found to be safe in a study where patients with DFU received 10 treatments in 2 weeks on consecutive business days. After treatment of 20 patients, the median decrease in wound size was 55%, and two wounds were completely healed after 2 weeks of treatment (Peters et al. 2017). The limited pain sensation reported by these DFU patients (slight pain in 5 out of 193 treatments) could be related to the underlying neuropathy leading to greatly diminished sensitivity (Volmer-Thole and Lobmann 2016). Before initiating a clinical study involving burn patients, we therefore determined safety and efficacy of the PLASOMA prototype in healthy volunteers. Subjects indicated that some transient, moderate pain was experienced during CAP treatment, with a mean score on the NRS of 3.3 (Table 1). All subjects rated the treatment acceptable despite several high NRS values. In studies where pain due to plasma treatment was scored with a visual analogue scale, a similar range of pain score (i.e. 0–5) was reported (Brehmer et al. 2015; Daeschlein et al. 2015b; Daeschlein et al. 2012a; Daeschlein et al. 2012b; Kisch et al. 2016a; Kisch et al. 2016b; Preissner et al. 2016).

Although referred to as cold or low temperature, CAP transiently increased the mean temperature of ex vivo skin with 9°C (Fig. 5) and of the skin in healthy volunteers with 3.4°C (Fig. 7a). The latter translates to an absolute skin temperature of 35.5°C ± 3.4°C after removal of the pad. The lower increase in skin temperature in volunteers due to CAP treatment is probably related to heat transfer to the surrounding tissues and via blood circulation. Importantly, higher pain scores did not correlate with higher skin temperatures. Similar results have been reported in other studies. Plasma increased the skin temperature with 2°C (Borchardt et al. 2017; Fluhr et al. 2012) or resulted in a sensation of heat score of 0–5 (Daeschlein et al. 2012a; Daeschlein et al. 2012b). Application of 42°C for 30 seconds could be well tolerated by volunteers (Oliveira et al. 2014) and stimulated blood flow, while fast heating resulted in a higher endothelial activity (Del Pozzi et al. 2016; Hodges et al. 2017). Natural cooling by exposure to ambient air quickly normalized blood flow (Wu et al. 2017). Natural cooling did not restore erythema index in our study, which indicates that this was not caused by the heat of the plasma. After applying external heat to the forearm of subjects, the mean thermal threshold for pain was approximately 47°C (Yosipovitch et al. 2004). In another study, pain at threshold temperature (median 44°C) was scored 4.5 (median NRS) (Wasner and Brock 2008). To thermally kill bacteria on the skin, a temperature of 60°C for 30 min is required (Tsuji et al. 1982). Since the increase in skin temperature was only 3.4°C (Fig. 7a) and treatment time was short, the effect of heat on bacterial inactivation can be excluded.

CAP application with the PLASOMA prototype induced a significant but transient increase in erythema index. The erythema index is proportional to the haemoglobin content of the upper layers of the dermis and thus might be considered an indirect measure for microcirculation, although in scars a correlation between microcirculation as measured by laser Doppler imaging and erythema was not found (Jaspers et al. 2017b). Plasma can stimulate microcirculation of healthy skin beyond the treatment time (Borchardt et al. 2017; Fluhr et al. 2012; Heuer et al. 2015; Kisch et al. 2016a; Kisch et al. 2016b), which can improve the healing potential especially of chronic wounds. The enhanced microcirculation is probably mediated by plasma-produced nitric oxide (NO) (Heuer et al. 2015), which easily penetrates the skin (Ganzarolli de Oliveira 2016; Vercelino et al. 2013). The potential role of plasma-generated NO in medicine is discussed by Suschek et al. (Suschek and Opländer 2016). NO is an important messenger and regulator of blood flow, immune response and wound healing, and NO can act as an anti-oxidant. Together these results on skin temperature and erythema suggest that the applied CAP induced a transient increase in microcirculation through the action of heating and possibly NO.

The barrier function of skin was probed by measuring the Trans Epidermal Water Loss (TEWL). Application of CAP or heat will result in vasodilation and increased TEWL. Because TEWL values were increased in both arms in our study, this was most likely related to the temporary occlusion by the pad and not to the CAP treatment. TEWL values returned to normal levels after 30 min in groups B and C, which was probably related to the swabbing and cleaning procedure used to determine bacterial survival. The reason for the sustained high TEWL levels in group A of our study remains elusive. Literature reports on TEWL related to plasma treatment are inconclusive: TEWL values were reduced by pulsed plasma jet and DBD by about 20% (Daeschlein et al. 2012a) or were increased by plasma jet with 50% (Fluhr et al. 2012) due to plasma treatment.

Plasma is highly bactericidal in vitro, which has been shown for various plasma sources in many papers. Several studies have tested the bactericidal effect of plasma generated by DBD (Daeschlein et al. 2012b; Li et al. 2013) or jet (Julák and Scholtz 2013; Lademann et al. 2012) devices for medical use on intact skin. Significant bacterial log reductions ranging from 0.3 to 2.7 were obtained after treatment times ranging from a few seconds to 10 min. After analysis of five clinical trials in a systematic review, CAP with DBD or jet demonstrated no significant reduction of bioburden in wounds (OR=0.85, *p*=0.63) (Assadian et al. 2019). Here we tested a DBD device on intact skin of volunteers that was contaminated with *P. aeruginosa*. We obtained a significant bacterial log reduction of 2.9 after a 1-min treatment. Bacteria on intact skin are more easily killed than bacteria in wounds, where blood, exudate and cellular material may limit the bactericidal effect especially when bacteria are present in a biofilm.

The power setting of the PLASOMA prototype did not affect the increase in temperature of ex vivo skin after CAP treatment. In healthy volunteers, the low power setting resulted in significantly less erythema and in trends towards lower skin temperature and less pain. Similar results were seen in mouse experiments where lowering the power of the plasma jet eliminated (or reduced) the temperature effect (Chatraie et al. 2018). Although lowering the power settings might reduce the disinfecting abilities of plasma devices (Zheng et al. 2016), the mean LR in our study was not significantly affected (Fig. 8).

This study has several limitations. We have treated only a small number of subjects, especially when comparing two power settings. Technical problems with this prototype device, i.e. device error or no plasma, occurred in many cases when treating volunteers and could be solved after repeated attempts to generate plasma. Device deficiency did not lead to a (S)AE; device errors were generated when the safety circuit of the device intervened. Nevertheless, short CAP applications were found safe and demonstrated good bactericidal properties.

## Supplementary Information

ESM 1 (PDF 476 kb)

## Data Availability

The datasets generated during and/or analysed during the current study are available from the corresponding author on reasonable request.
