# Supplementary material for: Antibacterial and safety tests of a flexible cold atmospheric plasma device for the stimulation of wound healing
Source: Appl Microbiol Biotechnol. 2021 Feb 15;105(5):2057–70. doi: 10.1007/s00253-021-11166-5 (PMC7906937; doi:10.1007/s00253-021-11166-5)
Supplement: Supplementary file 1 — (PDF 476 kb) [file 253_2021_11166_MOESM1_ESM.pdf]

Supplementary materials

Applied Microbiology and Biotechnology

Antibacterial and safety tests of a new flexible cold atmospheric plasma device for the stimulation of wound healing

Bouke Boekema[1], Matthea Stoop[2], Marcel Vlig[1], Jos van Liempt[3], Ana Sobota[3], Magda Ulrich[1,4,5], Esther Middelkoop[1,2,4]

[1]Association of Dutch Burn Centres, Beverwijk, The Netherlands
[2]Burn Center, Red Cross Hospital, Beverwijk, The Netherlands
[3]Eindhoven University of Technology, Department of Applied Physics, The Netherlands
[4]Amsterdam UMC, Vrije Universiteit Amsterdam, Department of Plastic, Reconstructive and Hand Surgery, Amsterdam Movement Sciences, Amsterdam, the Netherlands
[5]Amsterdam UMC, Vrije Universiteit Amsterdam, Department of Pathology, Amsterdam, the Netherlands

Correspondence: bboekema@burns.nl

Table S1 Demographic data of healthy volunteers

Group	N	Male	Female	Age[1] (y)	Fitzpatrick skin type			
					2	3	4	5
**A**	10	3	7	64 ± 9	4	5	1	0
**B**	8	5	3	52 ± 13	0	5	2	1
**C**	7	5	2	56 ± 13	1	4	2	0
**All**	25	13	12	58 ± 12	5	14	5	1

[1]The mean ± standard deviation is shown. Age was significantly higher in group A versus B (MWU).

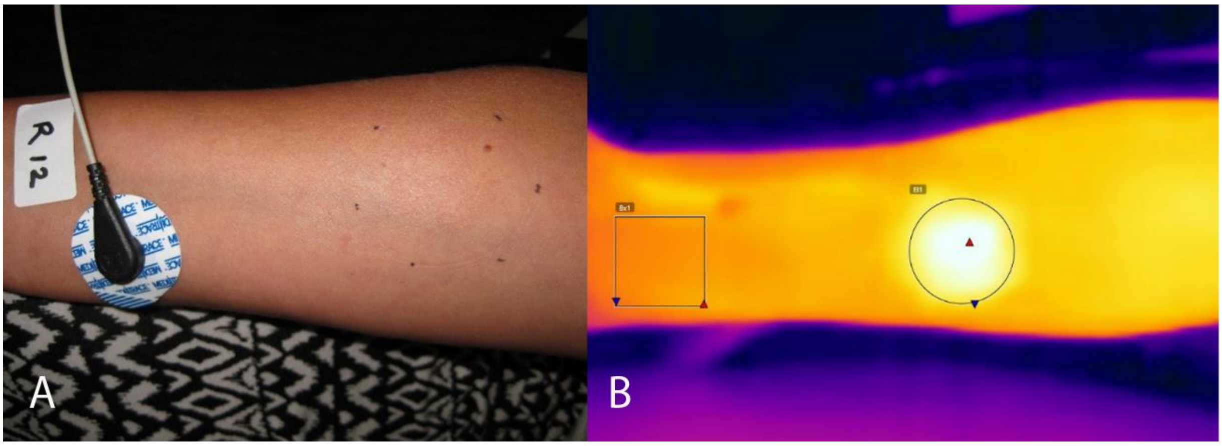

Figure S1 (**A**) Photograph showing the area of interest (black spots) on the inner forearm and reference electrode (ECG patch); (**B**) FLIR image directly after treatment and removal of the pad and reference electrode. The average and maximum temperatures were measured from the circular (treatment) and rectangular (reference) areas.
